# Changes in serum fatty acid and lipoprotein subclass concentrations from prepuberty to adulthood and during aging

**DOI:** 10.1007/s11306-016-0968-y

**Published:** 2016-02-08

**Authors:** Tarja Rajalahti, Chenchen Lin, Svein Are Mjøs, Olav Martin Kvalheim

**Affiliations:** Fjordomics, Førde Central Hospital, Førde, Norway; Department of Chemistry, University of Bergen, Bergen, Norway; Faculty of Health Studies, Sogn og Fjordane University College, Førde, Norway

**Keywords:** Human serum, Lipoprotein subclasses, Fatty acids (FAs), Docosahexaenoic acid (DHA), Eicosapentaenoic acid (EPA), Aging

## Abstract

Concentrations in serum were determined for 18 fatty acids (FAs) and 21 lipoprotein main and subclasses by chromatographic analyses and the average size was calculated for very low density (VLDL), low density (LDL) and high density (HDL) particles. 283 ethnic Norwegian children and adults from the rural Fjord region of Western Norway were compared with the objectives to reveal patterns and gender differences during the development from prepuberty to adulthood and during aging in adults. Both genders showed a large increase in eicosapentaenoic acid (EPA) and docosahexaenoic acid (DHA) from child to adult. Males, but not females, show a significant increase in most C16–C18 FAs from prepuberty to adulthood. These changes in males correlate to a pattern of increased concentrations of triglycerides, VLDL and LDL particles, especially the atherogenic subclasses of small and very small LDL particles. Furthermore, concentrations of medium, large and very large HDL particles decrease, while concentration of very small HDL particles increase leading to reduced average size of HDL particles. Females only showed significant increase in concentrations of small and very small LDL particles, very small HDL particles and apolipoprotein B. While EPA and DHA continued to increase during aging in women, no validated model for connecting age to FA profile was obtained for men. Women showed significant increase in concentrations of all subclasses of LDL particles during aging, while men exhibited a more complex pattern with increase also in apolipoprotein A1 and HDL particles.

**Electronic supplementary material:**

The online version of this article (doi:10.1007/s11306-016-0968-y) contains supplementary material, which is available to authorized users.

## Introduction

Following the work of Dyerberg et al. (1978) and Kagawa et al. (1982), which indicated the protective effect of EPA on risk of developing cardiovascular diseases (CVDs), several studies have been performed to assess effects of FAs on lipoprotein distribution and their impact on CVD risk. Reviews by Chowdhury et al. (2014) and Michas et al. (2014) summarize the current opinion of the associations of individual and groups of FAs to CVD risk. The poly-unsaturated FAs, especially the marine omega-3 FAs (Kelley and Adkins 2012; Ninomiya et al. 2013), appear to reduce CVD risk, while saturated and mono-unsaturated FAs increase CVD risk. Similarly, certain lipoprotein subclasses have been shown to be connected to increased CVD risk, e.g., small LDL particles (Hirayama and Miida 2012).

Lin et al. (2016) used multivariate analysis to reveal relations between lipoprotein subclasses and FA patterns in a cohort of healthy Norwegians. A limitation in that study was that only adults were included. Thus, it was not possible to investigate changes in lipoprotein and FA patterns accompanying the evolution from child to grown-up. However, age effects on FA levels have been studied by Risé et al. (2013). They observed changes in relative levels of EPA and DHA in blood from child to adult and to elderly in both genders. Otsuka et al. (2013, 2015) independently found positive correlations between age and absolute concentrations of EPA and DHA in adults aged 40–79 years. Harris et al. (2013) analyzed red blood cells of 160,000 patients that were examined for cardiovascular (CV) risk factors and observed an increase in relative concentrations of EPA and DHA.

Age-related effects have also been observed for lipoproteins. Total cholesterol levels as well as HDL and LDL levels peak around the age 9–10 years for children and then decrease during adolescence before increasing again from the age 16–17 (Labarthe et al. 2003; Kaitosaari et al. 2009; Dai et al. 2009). By the end of puberty the gender dependent patterns of lipoprotein cholesterol are evident (Stozicki et al. 1991; Freedman et al. 2001). Less is known about aging effects on lipoprotein patterns in adults, but Freedman et al. (2004) observed impact on some lipoprotein subclasses.

Our objectives with this investigation are to disclose patterns of change, from the prepubertal cholesterol peak in children to adulthood, and during aging, with possible implications for cardiovascular health, hidden in the concentration profiles of serum FAs and lipoprotein subclasses. Furthermore, to explore gender differences in the alterations of both FAs and lipoprotein features accompanying the development from child to adult and through the aging process. Multivariate discriminant (Sjöström et al. 1986; Rajalahti et al. 2010) and regression analyses (Wold et al. 1984) are used as tools to search for patterns.

## Materials and methods

### Participants

A cohort of 10 years old healthy ethnic Norwegian children, 91 boys and 56 girls, and 136 healthy ethnic Norwegian adults, 69 women (age 40 (mean) ± 11 (sd) and 67 men (age 41 ± 11), were recruited in the rural Fjord region of Western Norway. Approximately half of the children and adults were recruited in 2011 (Batch A) and half in 2014 (Batch B). A detailed description of the adult cohort can be found in Lin et al. (2016).

### Blood sampling

Blood samples were collected between 8 and 10 am after overnight fasting. Serum was obtained according to the standardized protocol described in Lin et al. (2016). Serum was split into 0.5 ml aliquots and stored in cryotubes at −80 °C.

### Measurement of fatty acid profile

The serum samples were prepared and FAs quantified by use of chromatography as described in Lin et al. (2016). The samples from both surveys were extracted and analyzed in random order. The total amounts of FAs in each serum sample were converted into amounts in μg per g sample by dividing with the sample weights. The medians of the 18 FAs for children and adults were calculated for both genders (Supplementary material 1). This dataset includes the majority of FAs that are considered biologically important. Total fatty acid (TFA) concentration and the ratio of EPA to arachidonic (AA), i.e. EPA/AA, are also included in supplementary material 1. Systematic names and abbreviations to common names defined in text are used. See supplementary material 3 for Chebi Ids.

### Measurement and features calculated for lipoproteins

Serum lipoproteins were analyzed on an HPLC system at Skylight Biotech (Akita, Japan) according to the procedure based on high-performance liquid chromatography (HPLC) analysis and curve fitting to define lipoprotein subclasses (Usui et al. 2002; Okazaki et al. 2005). Two pools of subjects, i.e. normolipidemics and hypertriglyceridemics, were used to establish 20 lipoprotein subclasses. Within-day precision (n = 5) for the two pools determined as coefficient of variation (CV) was 0.2–4.4. The samples were analyzed in two rounds, one in 2011 and one in 2014. Replicates of five serum samples analyzed in 2011 (Batch A) were included in the batch B from 2014 in order to be able to detect and correct for possible systematic differences between batches.

Using the procedure described in Lin et al. (2016), the following lipoprotein features were calculated: Concentrations of total cholesterol (Chol), triglyceride (TG), chylomicrons (CM) (>80 nm), VLDL (30–80 nm), LDL (16–30 nm) and HDL (8–16 nm) particles. Range of particle size is provided in parentheses. Furthermore, concentrations of 4, 4 and 5 subclasses of VLDL, LDL and HDL particles, respectively, labeled as VLDL-VL (64, and, 53.6), VLDL-L (44.5). VLDL-M (36.8), VLDL-S (31.3), LDL-L (28.6), LDL-M (25.5), LDL-S (23.0), LDL-VS (20.7, 18.6, and, 16.7), HDL-VL (15.0, and, 13.5), HDL-L (12.1), HDL-M (10.9), HDL-S (9.8) and HDL-VS (8.8, and, 7.6). The abbreviations VL, L, M, S and VS denote very large, large, medium, small and very small particles, respectively. The average particle size, in nm, is provided in parentheses for each of the 20 subclasses defining the 13 subclasses calculated in this work. Where subclasses are combined, the average diameter of each of the original subclasses is provided. In addition, average particle size of each of the main lipoprotein classes VLDL, LDL and HDL was estimated. Note that the subclass VLDL-S is often labeled as intermediate density lipoprotein (IDL).

Serum apolipoproteins A1 (ApoA1) and B (ApoB) were measured by turbidimetric immunoassay using commercially available kits (Sekisui Medical co., Ltd, Tokyo, Japan). The serum samples for the children recruited in the second round were not analyzed for ApoA1 and ApoB.

Medians for all the lipoprotein features for children and adults of both genders are provided in supplementary material 2.

### Removal of systematic batch differences in the lipoprotein data

As explained above, the lipoprotein analyses were performed in two rounds by HPLC followed by curve resolution and calculation of main and subclass concentrations and average particle size for VLDL, LDL and HDL. The second batch of samples was analyzed approximately 2 years after the analysis of the first batch. In order to be able to detect possible systematic differences between the two batches, five samples from the first batch (A) were reanalyzed together with the samples from the second batch (B). The profiles of these five replicated samples revealed relatively large systematic differences in some lipoprotein features between the two batches. The concentrations of VLDL and its subclass VLDL-L were higher and its subclass VLDL-S lower in the second batch. For LDL, the picture was opposite with concentrations of LDL and its subclasses LDL-L and LDL-M being lower and LDL-S and LDL-VS being larger in the second batch. These shifts lead to increased average size of VLDL and decreased average size of LDL particles in the second batch compared to the first batch. For each of the lipoprotein features showing systematic differences, the batch difference was calculated for each replicate and the measurements of all samples in the second batch were adjusted by adding the median of these differences. This procedure assumes that the five replicates are representative for the analytical differences between batches and that the effect is additive. After this preprocessing, termed median difference correction (MDC), pairs of replicates coincided in the principal component (PC) score plots (Jolliffe 1986) on the major PCs confirming that the systematic difference had been eliminated.

### Data analysis

The hypothesis of equal medians in the FAs and lipoproteins for the children and the adults were tested for both genders using Bonferroni corrected values of the Wilcoxon-Mann–Whitney (WMW) nonparametric rank sum test (Wilcoxon 1945; Mann and Whitney 1947). Matlab R2013b was used for calculation (MathWorks, Natick, Massachusetts, U.S.A). Furthermore, false discovery rates (Benjamini and Hochberg 1995) were calculated.

Multivariate data analysis was performed by means of the commercial software Sirius Version 10.0 (Pattern Recognition Systems AS, Bergen, Norway). Prior to multivariate analysis, all variables were centered and standardized to unit variance. Preprocessing to unit variance was done due to large differences in variance between variables. Partial least squares discriminant analysis (PLS-DA) (Sjöström et al. 1986) was used to reveal discriminating features in FA and lipoprotein patterns between children and adults for both genders. The FAs (Supplementary material 1) and lipoprotein features (Supplementary material 2) were modelled separately, thus giving rise to four PLS-DA models. Repeated double cross validation (RDCV) (Westerhuis et al. 2008) was used for optimizing the predictive performance of all models. 100 repetitions were performed with 10 % of the samples in outer loop and seven partitions in the inner loop. Mean and standard deviations for RMSEP and Q2Y were calculated for each PLS component and the number of PLS components were determined when the value of Q2Y minus two standard deviation exceeded the previous Q2Y. All PLS-DA models were further validated by a randomization test whereby elements in the y-vector were exchanged and the Q2Y from the true model was compared with the distributions from 1000 permutations. A p-value was calculated from the number of permuted models with Q2Y exceeding Q2Y of the true model. Also for this test, 10 % of the samples were randomly kept in outer loop until all samples had been kept out once for each permutation. Percent correct classification rate (%CCR) was finally calculated as an additional performance test of the discriminatory ability of the PLS-DA models. Also for this calculation, prediction was performed by keeping out 10 % of the samples in the outer loop. Table 1 summarizes the performance measures for each model.

**Table 1:** Performance measures calculated for all models. A and B in parentheses imply samples from subjects recruited in the first (A) and second (B) round, respectively

Model	R2Y	Q2Y	% CCR	p value
Girls & women (A + B), FAs	0.63	0.48 ± 0.03	86	0.001
Boys & men (A + B), FAs	0.65	0.57 ± 0.02	89	0.001
Women (A + B), age = f(FAs)	0.36	0.19 ± 0.04		0.007
Men (A + B), age = f(FAs)	No validated model			
Girls & women (A + B), lipoproteins	0.36	0.24 ± 0.04	72	0.016
Boys & men (A + B), lipoproteins	0.64	0.59 ± 0.01	88	0.001
Women (A + B), age = f(lipoproteins)	0.32	0.22 ± 0.02		0.001
Men (A + B), age = f(lipoproteins)	0.40	0.28 ± 0.03		0.001
Girls & women (A), FAs	0.64	0.37 ± 0.09	84	0.029
Girls & women (B), FAs	0.58	0.35 ± 0.03	80	0.029
Boys & men (A), FAs	0.67	0.49 ± 0.07	82	0.009
Boys & men (B), FAs	0.63	0.52 ± 0.24	86	0.004
Women (A), age = f(FAs)	0.37	0.16 ± 0.07		0.033
Women (B), age = f(FAs)	0.75	0.25 ± 0.05		0.002
Girls & women (A), lipoproteins	0.68	0.37 ± 0.06	90	0.034
Girls & women (B), lipoproteins	0.25	0.13 ± 0.02	69	0.064
Boys & men (A), lipoproteins	0.69	0.56 ± 0.02	90	0.008
Boys & men (B), lipoproteins	0.65	0.57 ± 0.03	89	0.005
Women (A), age = f(lipoproteins)	0.43	0.23 ± 0.09		0.001
Women (B), age = f(lipoproteins)	No validated model			
Men (A), age = f(lipoproteins)	0.38	0.20 ± 0.08		0.019
Men (B), age = f(lipoproteins)	0.54	0.36 ± 0.06		0.001

Calculation of Q2Y and % correct classification rate (% CCR) is based on RDCV (Westerhuis et al. 2008) with 10 % of samples in outer loop for predictions and 100 repetitions. Mean and confidence limits corresponding to two standard deviations are provided. The p values are calculated from randomization tests with 1000 permutations and RDCV with 10 % of subjects in outer loop

For each validated PLS model, a single predictive component was calculated by means of target projection (TP) (Kvalheim and Karstang 1989; Rajalahti and Kvalheim 2011). Selectivity ratios (SRs) (Rajalahti et al. 2009a, b) were obtained as the ratio of explained to residual variance for each FA (or lipoprotein feature) on the predictive (target) component. By multiplying the SRs with the sign of the corresponding loading on the predictive component, an SR plot was constructed. This plot displays the FAs (or lipoprotein features) according to their discriminatory importance for the model. From the RDCV procedure, confidence bounds can be constructed around each SR value and used to assess the significance of each variable separately. By using the same procedure to calculate rank sums for two groups of subjects as in the WMW rank sum test for unpaired measurements of two groups, we can obtain a rank sum classification rate (RSCR) for each variable. Every variable is sorted from smallest to largest and given a rank starting from one for the smallest value and increasing in step by one until the largest one which gets a rank equal to the number of samples. We can then calculate a summed rank for each group that can be compared with the minimum possible rank in each group corresponding to perfect classification. From these numbers, the RSCR can be calculated. Subjects from one group that are located far into the other group reduce RSCR more than subjects that are border-line. The RSCR can be used to connect SR values to the univariate WMW test statistics and to the corresponding univariate classification performance.

Standard partial least squares (PLS) regression models (Wold et al. 1984) for adults with age as y-variable were then created for both genders based on either the FAs (supplementary material 1) or the lipoprotein features (supplementary material 2). Performance measures for these models are provided in Table 1. SR plots were obtained using the same procedure as for the PLS-DA models. These plots display and rank the FAs and the lipoprotein features according to their importance for predicting age in adults.

As an additional validation of the results, the above procedure was repeated with separate modeling of the two batches of samples. The results are presented in Table 1 above. Hierarchical clustering (HCA) with average linkage for clustering and Euclidean distance as metric (Kaufman and Rousseeuw 1990) was subsequently employed with SR profiles as input to assess similarity between models based on batch A, batch B and the combination of A and B.

## Results and discussions

### Differences in medians between children and adults

Previous work (Freedman et al. 2004; Johnson et al. 2004; Lin et al. 2016) has shown large gender differences in both FA and lipoprotein patterns. Thus, males and females have to be analyzed separately. Supplementary material 1 shows medians of FAs, TFA and EPA/AA calculated for children and adults of both genders. Furthermore, p-values from Wilcoxon-Mann–Whitney (WMW) unpaired rank sum test are shown for a comparison of medians between children and adults for both genders. These p-values have to be corrected for multiple testing. If we assume that the 20 FA features constitute the same family of tests and use the Bonferroni correction, the p_WMW_-values have to be less or equal to 0.0005 to be significant for p_Bonferroni_ = 0.01. After this correction, only EPA, DHA, EPA/AA and nervonic acid (24:1 n-9) show significant differences in the medians of prepubertal and adult females, while docosapentaenoci acid (DPA) is just on the borderline of significance.

Comparison of prepubertal and adult males presents a different picture. Only for seven FAs are the medians not significantly different after Bonferroni correction. The borderline is drawn between nervonic acid (significant) and 22:0 (not significant). The most significant differences are found for DHA, DPA, 18:1 n-7, 16:1 n-7, EPA and EPA/AA with Bonferroni corrected p-values in the range 2.8 × 10^−12^–6.8 × 10^−9^.

If we instead use false discovery rate (FDR) (Benjamini and Hochberg 1995) for assessment of significance, then p_WMW_ = 0.0075 corresponds to p_FDR_ = 0.01 and also the medians for the FAs 22:0 and 14:0 are significantly different between boys and men. The same kind of approach applied for comparison of medians in girls and women adds DPA, 24:0 and linoleic acid (LA) as significantly different at p_FDR_ = 0.01 which corresponds to p_WMW_ = 0.0035.

Thus, both genders experience significant increase in concentrations of EPA, DHA and EPA/AA from prepuberty to adulthood, but males show pronounced increase also in many other FAs. The lower concentrations of EPA and DHA in children compared to adults may, however, imply that these two crucial FAs are utilized at a higher rate during childhood with rapid development of brain and body (Risé et al. 2013). Overnight fasting before sampling may thus explain why EPA and DHA are low in children´s serum. An alternative explanation is that the children’s dietary intake is different from the adults’, the two groups being separated by, on the average, 30 years in age. Since children and adults live in the same Fjord region and are influenced by the same food culture, this explanation seems less plausible than the biological hypothesis of attributing the difference to growth versus maintenance of brain and body.

Supplementary material 2 shows the medians of main and subclasses of lipoproteins and the average particle size of VLDL, LDL and HDL for children and adults of both genders together with p-values comparing children with adults using the WMW unpaired rank sum test. Regarding all the 24 features as belonging to one family of test, the borderline corresponding to p = 0.01 for Bonferroni corrected p-values, is p_WMW_ = 4.2 × 10^−4^. For females, only the medians of the subclasses of very small and small LDL particles are significantly different between pre-puberty girls and women. They have p_Bonferroni_-values of 1.6 × 10^−5^ and 1.5 × 10^−3^, respectively. The median of very small HDL particles is just on the borderline of significance with p_Bonferroni_ = 1.3 × 10^−2^. For males the picture is very different. Only the medians of small HDL particles and ApoA1 are not significantly different at p_Bonferroni_ = 0.01.

Using the approach of FDR does not change the picture. Thus, p_FDR_ = 0.01 corresponds to p_WMW_ = 0.00125 and the changes in median for very small HDL particles unites with small and very small LDL particles as significantly different for prepubertal and adult females. For males, p_FDR_ = 0.01 corresponds to p_WMW_ = 0.00958 and ApoA1 adds as significant for males. In summary, large changes in lipoprotein distribution are observed for males, while only few significant changes are revealed for females from prepuberty to adulthood.

### Changes in FA pattern during aging

**Fig. 1:**
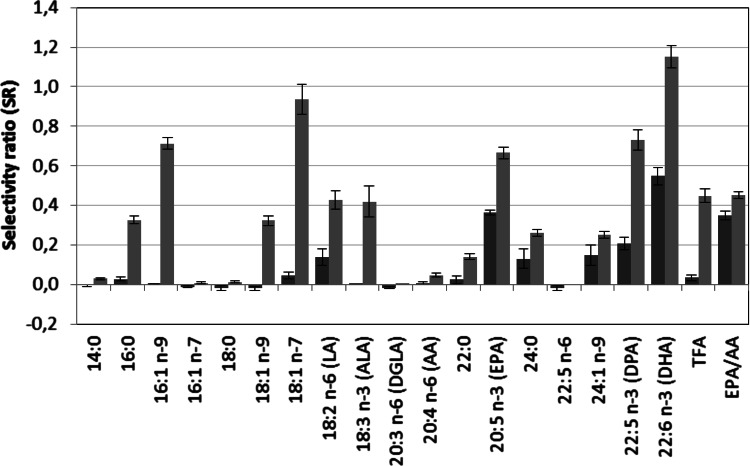
SR plot displaying changes in fatty acid patterns from prepuberty to adulthood for females (*red*), and, males (*blue*). Confidence limits correspond to two standard deviations (Color figure online)

PLS-DA was used to investigate alterations of FA profiles in males and females from prepuberty to adulthood. Strong discriminatory models (Table 1) could be established for both males and females using age group as dependent variables (children = 0, adults = 1). In order to reveal which features were responsible for the discrimination in FA profiles between children and adults, target projection (TP) was performed to obtain a single discriminatory component for each model. From this component selectivity ratios (SRs) were calculated for all features and presented as SR plots as shown in Fig. 1. Confidence limits are calculated from the RDCV procedure and plus and minus sign indicates increase or decrease from prepuberty to adulthood and corresponds to positive or negative loadings on the discriminatory (target) component. The SR plot reveals clear gender differences. For females (Fig. 1, red color) the largest changes from prepuberty to adulthood are observed for DHA (SR = 0.55), EPA (SR = 0.36), and EPA/AA (SR = 0.35). Concentrations of DPA (SR = 0.21), nervonic acid (SR = 0.15), LA (SR = 0.14) and 24:0 (SR = 0.13) show small changes between children and adults. All the other FAs have SR close to zero and are thus unaltered in concentrations from prepuberty to adulthood in females. By calculating the rank sum classification rate (RSCR). it is possible to relate the discriminatory ability of individual FAs to SR. While RSCR is 77 % for the most discriminating FA, namely DHA, it is reduced to 67 % for DPA. These observations are in line with the results from comparison of medians from the Bonferroni corrected WMW-tests discussed above. The observed age-related increase in absolute DHA and EPA concentrations complies with other investigations published recently (Risé et al. 2013; Otsuka et al. 2013, 2015; Harris et al. 2013).

For males the pattern of change in FAs are more complex than for females. The SR plot (Fig. 1, blue color) reveals changes also in many of the C14–C18 FAs. Thus, in addition to similar changes as for females with increase in serum levels of DHA (SR = 1.2), DPA (SR = 0.7), EPA (SR = 0.7) and EPA/AA (SR = 0.5), also C14–C18 FAs increase in concentrations. In particular, 18:1 n-7 (SR = 0.9) and 16:1 n-9 (SR = 0.7) increase in males, but also some of the other C14–C18 FAs show significant increase such as LA (SR = 0.4) and α-linolenic acid (ALA), 18:3 n-3, (SR = 0.4). DHA, DPA and EPA all provide RSCR larger than 82 % underlining their significant increase in concentration from prepuberty to adulthood in males.

We have so far only considered the changes in FA profiles between children and adults, but the cohort of adults have a span in age of approximately 40 years with a mean of 40. Although both genetic factors and lifestyle with respect to diet and level of physical activity impact on the FA profiles of individual subjects (Aadland et al. 2013), it is worth trying to associate features of the FA profiles to age in order to examine if the increase in serum levels of the marine omega-3 FAs observed between children and adults may represent a continuous process accompanying aging.

**Fig. 2:**
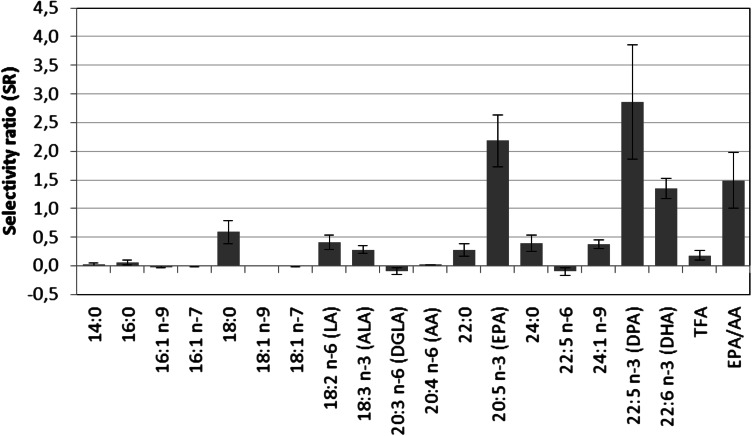
SR plot displaying changes in fatty acid pattern during aging for women. Confidence limits correspond to two standard deviations

It was not possible to obtain a validated multivariate model for men, but for women a validated PLS model with weak, but significant predictive ability was obtained. The model performance measures (Table 1) reflect, as expected, that variation in age alone cannot account for the variation in the FA profiles. The root mean square error of prediction (RMSEP) of 10 ± 0.3 years confirmed this. The SR plot (Fig. 2) is dominated by large increase in the marine omega-3 FAs: DPA (SR = 2.9), EPA (SR = 2.2), DHA (SR = 1.4) and the ratio EPA/AA (SR = 1.5). These are the same FAs that dominated the model comparing children with adults except that DPA seems to increase more than EPA and DHA with age in the adult female cohort. Our conclusions complies with results from previous investigations using univariate statistics to test for age-related changes in EPA, DHA and EPA/AA (Otsuka et al. 2013, 2015; Harris et al. 2013; Risé et al. 2013),

### Changes in lipoprotein pattern during aging

PLS-DA with age group as dependent variables (children = 0, adults = 1).was subsequently used to investigate alterations of lipoprotein features in males and females from prepuberty to adulthood. The changes in lipoprotein pattern are much more pronounced in males than in females (Supplementary material 2).

**Fig. 3:**
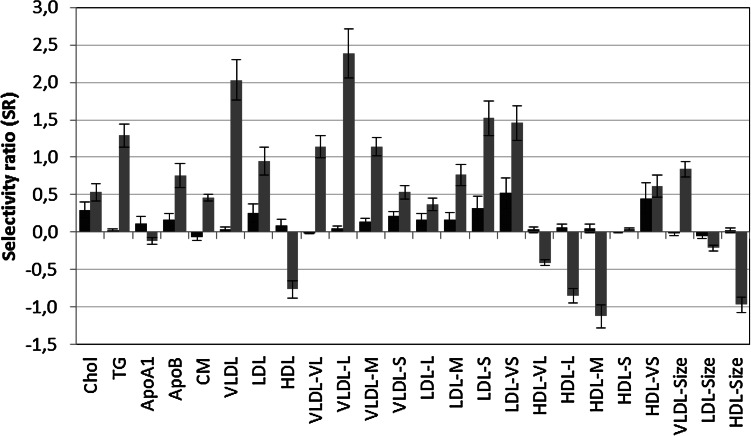
SR plot displaying changes in lipoprotein patterns from prepuberty to adulthood for females (*red*), and, males (*blue*). Confidence limits correspond to two standard deviations (Color figure online)

SR plot for both models is shown in Fig. 3 and reveals distinct gender differences in patterns. Lipoprotein features in females (Fig. 3, red color) seem to undergo small changes from prepuberty to adulthood. Increase in concentrations of the very small (SR = 0.52) and small (SR = 0.33) LDL particles and very small HDL (SR = 0.45) particles are the most striking features followed by increase in total concentration of cholesterol (SR = 0.30) and LDL particles (SR = 0.26). RSCR is larger or equal to 72 % for all these features. This shows a shift towards a more atherogenic pattern in females, but the change is small, in line with the conclusions from the univariate tests in Sect. 3.1. As for the FA profiles, males (Fig. 3, blue color) show a more complex pattern of change in lipoprotein features from prepuberty to adulthood than females, and, unfortunately, to a more atherogenic pattern. Thus, large increase is observed for serum concentrations of TG (SR = 1.3), ApoB (SR = 0.8), VLDL (SR = 1.9), and LDL (SR = 1.0), and their subclasses VLDL-VL (SR = 1.2), VLDL-L (SR = 2.5), VLDL-M (SR = 1.2), LDL-S (SR = 1.5) and LDL-VS (SR = 1.4). Furthermore, total concentrations of HDL (SR = −0.8) together with the subclasses HDL-VL (SR = −0.4), HDL-L (SR = −0.8), and HDL-M (SR = −1.2), and thus the average size of HDL particles, HDL-Size (SR = −1.08), are reduced from childhood to adulthood, while the suspected atherogenic subclass (Freedman et al. 1998; Lin et al. 2016) HDL-VS (SR = 0.6) increases. RSCR is larger or equal to 82 % for both the atherogenic subclasses LDL-S and LDL-VS and their associate ApoB. This pattern implies a decline in cardiovascular (CV) health for males from prepuberty to adulthood that was only weakly present in females. These changes point to increased CVD risk for males compared to females (Furusyo et al. 2013 and refs. therein). These observations comply with the increase in C14–C18 FAs for males observed in Fig. 1 since we have found significant associations between C14 and C18 FAs and these lipoprotein features in a previous investigation (Lin et al. 2016).

**Fig. 4:**
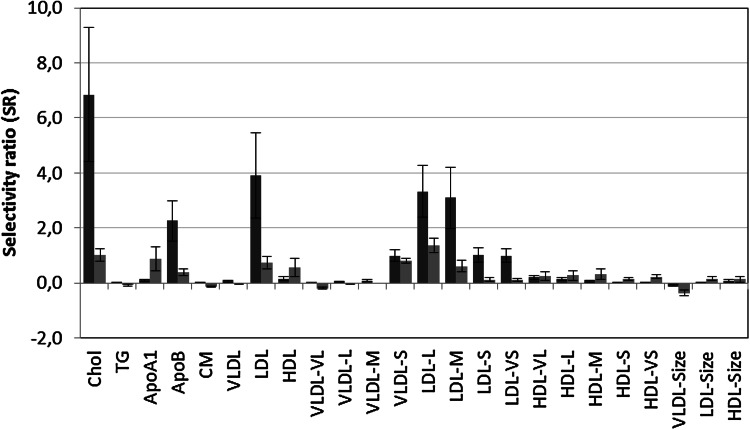
SR plot displaying changes in lipoprotein pattern during aging for women (*red*), and, men (*blue*). Confidence limits correspond to two standard deviations (Color figure online)

Multivariate PLS models were subsequently constructed for associating lipoprotein profiles to age for the adult cohort with performance characteristics as displayed in Table 1. RMSEP was 10.1 ± 0.2 years for women and 9.6 ± 0.2 years for men showing that other factors than age have large impact on the lipoprotein profile, The SR plot for women (Fig. 4, red color) reveals increase in concentrations of total cholesterol (SR = 6.9), ApoB (SR = 2.3), LDL (SR = 3.9), the subclass of small VLDL (SR = 1.0) and all subclasses of LDL particles, i.e. large (SR = 3.3), medium (SR = 3.1), small (SR = 1.0) and very small (SR = 1.0) LDL particles with age. Thus, although the change from prepuberty to adulthood is smaller for females than for males there is a progressive development towards a more atherogenic lipoprotein pattern during aging also in the adult female cohort.

The SR plot for men (Fig. 4, blue color) with changes in lipoprotein pattern accompanying increased age is different from women. Although many of the dominating features are the same, such as increase in concentrations of total cholesterol (SR = 1.0), ApoB (SR = 0.4) and LDL (SR = 0.7) and the subclasses of small VLDL (SR = 0.8) and, large (SR = 1.4) and medium (SR = 0.6) LDL particles, the relative age-related changes are smaller as reflected in the smaller SR values, but from higher concentration levels after puberty compared to women. There are also opposing features such as increase in concentrations of “healthy” lipoproteins such as HDL (SR = 0.6) and ApoA1 (SR = 0.9) and almost no changes in concentrations of the atherogenic small (SR = 0.1) and very small (SR = 0.1) LDL particles. These observations underline that other factors, than just age, must be important to explain the changes in lipoprotein pattern in adult males.

### Validation of results using HCA of models from separate batches

In order to validate results, separate models were calculated for the two batches (Table 1). For FAs, models with almost the same performance were obtained for each batch separately as for the combined batches. For lipoproteins, the low number of women in batch B, only 24, caused problems both for the models comparing patterns in girls and women and for predicting age in adults. This is expected since the lipoprotein patterns in girls and women are relatively similar as shown by the rather ow Q2Y and %CCR also for the model of the combined batches. Furthermore, predicting age for women from only 24 subjects, spanning a range of 20–60 years and with samples being spent in outer and inner loop for validation, leaves too few subjects for modelling and no validated model is obtained.

**Fig. 5:**
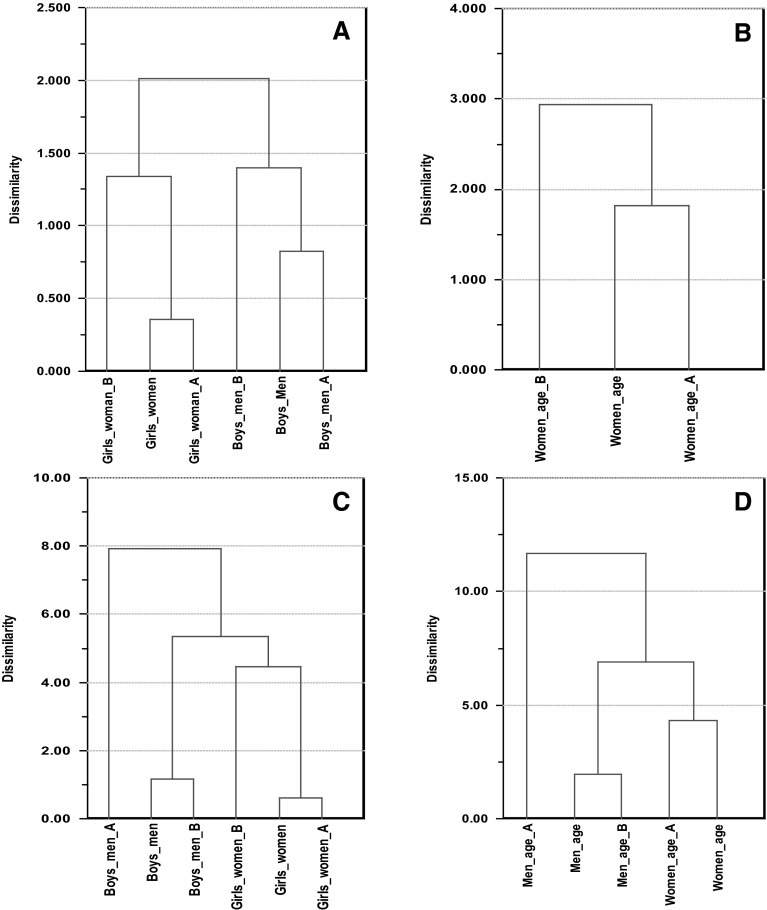
Dendrograms from hierarchical clustering analysis (HCA) showing similarities between SR patterns calculated from PLS-DA/TP models for each batch (A and B) of samples separately and the combination of both batches for **a** FAs comparing children and adults, **b** Age for women predicted from FAs, **c** Lipoprotein features comparing children and adults, and, **d** Age for both genders predicted from lipoprotein profiles. Note that it was not possible to obtain a validated model for predicting age from lipoproteins for batch B for women; probably due to the low number of subjects (24) representing an age range of 20–60

As a last step in the validation, we used hierarchical cluster analysis (HCA) to compare models on the basis of their SR pattern (Fig. 5). FA models are more similar than lipoprotein models, and, for the discriminatory models, males and female models group in separate clusters. For change of lipoprotein pattern with age, no FA model was possible for men, but for females, the dendrogram shows that the SR patterns are quite similar. For lipoproteins, batch A breaks the pattern by being different from batch B and the models combining batch A and B for males. PCA with SR profiles as input showed that batch A had larger increase in small and very small LDL particles and apoB than batch B from prepuberty boys to men and that increase in cholesterol had a much larger impact in the age model for men from batch A than batch B.

## Concluding remarks

We have shown that, for both genders, the absolute concentrations of serum EPA and DHA increase from prepuberty to adulthood. For women the process continues during aging, but we were not able to validate this for men. For males most C16–C18 FAs also increase from prepuberty to adulthood, but this was not observed for females.

The lipoprotein pattern changes to a more atherogenic pattern for males during the transformation from prepuberty to adulthood, while changes are much less pronounced for females. However, females show increase in atherogenic small and very small LDL particles. During the aging process in adults, the relative changes in lipoprotein pattern with impact on CV health appear more pronounced in woman than men, but women start from a much more favorable pattern after puberty than men. Thus, the gender differences in CV health narrow with age, but the mortality rate from coronary heart disease (CHD) among women never exceeds that of men (Freedman et al. 2004 and references therein).

## Electronic supplementary material

Supplementary material 1 (DOCX 15 kb)

Supplementary material 2 (DOCX 16 kb)
