# Supplementary material for: Changes in serum fatty acid and lipoprotein subclass concentrations from prepuberty to adulthood and during aging
Source: Metabolomics. 2016 Feb 8;12:51. doi: 10.1007/s11306-016-0968-y (PMC4744832; doi:10.1007/s11306-016-0968-y)
Supplement: Supplementary file 1 — Supplementary material 1 (DOCX 15 kb) [file 11306_2016_968_MOESM1_ESM.docx]

Supplementary material 1. Univariate statistical measures calculated for fatty acids for the children and adults. Medians are given in units of μg per g sample. p_WMW_ are the p-values calculated from the nonparametric Wilcoxon-Mann-Whitney (WMW) rank sum test (Wilcoxon 1945; Mann and Whitney 1947).

Variable Median Median

Girls Women p_WMW_ Boys Men p_WMW_

(N=56) (N=69) (N=91) (N=67)

**14:0** 36.6 37.8 0.818 36.0 40.9 0.005

**16:0** 790.9 839.6 0.062 754.2 907.2 6.8*10^-8^

**16:1 n-9** 14.5 13.6 0.521 12.4 17.3 5.0*10^-12^

**16:1 n-7** 75.9 72.1 0.424 65.3 67.5 0.529

**18:0** 304.1 283.3 0.317 297.7 304.2 0.167

**18:1 n-9** 778.8 708.3 0.213 726.6 896.1 4.8*10^-7^

**18:1 n-7** 50.5 53.2 0.075 45.9 60.5 1.5*10^-12^

**18:2 n-6 (LA)** 1054.8 1214.7 0.002 1047.7 1248.4 2.5*10^-7^

**18:3 n-3 (ALA)** 24.5 26.4 0.439 23.0 34.9 3.0*10^-8^

**20:3 n-6 (DGLA)** 59.9 54.4 0.084 60.3 60.1 0.986

**20:4 n-6 (AA)** 250.1 244.7 0.814 245.3 279.8 0.040

**22:0** 35.6 36.8 0.306 33.6 37.6 0.003

**20:5 n-3 (EPA)** 29.8 52.3 1.5*10^-7^ 28.2 55.6 3.6*10^-11^

**24:0** 32.5 36.6 0.002 32.0 38.6 6.2*10^-6^

**22:5 n-6** 5.5 4.2 0.034 5.3 5.0 0.288

**24:1 n-9** 54.3 63.4 3.7*10^-4^ 52.7 63.1 1.4*10^-5^

**22:5 n-3 (DPA)** 28.7 32.0 6.7*10^-4^ 27.5 35.4 3.4*10^-13^

**22:6 n-3 (DHA)** 81.6 118.1 6.3*10^-8^ 72.6 120.6 1.4*10^-13^

**TFA** 3873.5 3896.3 0.104 3522.9 4284.5 1.7*10^-8^

**EPA/AA** 0.120 0.208 5.9*10^-7^ 0.113 0.225 3.4*10^-10^
