# Supplementary material for: Changes in serum fatty acid and lipoprotein subclass concentrations from prepuberty to adulthood and during aging
Source: Metabolomics. 2016 Feb 8;12:51. doi: 10.1007/s11306-016-0968-y (PMC4744832; doi:10.1007/s11306-016-0968-y)
Supplement: Supplementary file 2 — Supplementary material 2 (DOCX 16 kb) [file 11306_2016_968_MOESM2_ESM.docx]

Supplementary material 2. Medians of lipoprotein features for the pre-puberty girls and boys and women and men. Median concentrations are given in units of mg per dl serum, while particle size is given as nm. p_WMW_ are the p-values calculated from the nonparametric Wilcoxon-Mann-Whitney (WMW) rank sum test (Wilcoxon 1945; Mann and Whitney 1947).

Variable Median Median

Girls Women p_WMW_ Boys Men p_WMW_

(N=56) (N=69) (N=91) (N=67)

**Chol** 160.9 176.2 0.008 161.7 196.9 9.1*10^-9^

**TG** 57.8 63.4 0.158 49.0 96.4 9.9*10^-15^

**CM** 1.0 1.4 0.776 0.8 3.6 1.4*10^-12^

**VLDL** 59.1 61.0 0.262 43.6 97.6 1.3*10^-16^

**LDL** 100.0 110.0 0.007 99.5 135.7 1.7*10^-12^

**HDL** 64.3 69.5 0.029 67.2 55.3 1.1*10^-11^

**VLDL-VL** 11.0 10.5 0.872 7.2 26.6 4.5*10^-14^

**VLDL-L** 18.0 18.6 0.306 11.4 32.0 1.0*10^-16^

**VLDL-M** 13.9 15.9 0.103 11.6 21.4 1.3*10^-13^

**VLDL-S** 13.4 14.9 0.018 12.9 17.7 1.1*10^-9^

**LDL-L** 38.9 41.8 0.027 39.4 48.4 4.3*10^-7^

**LDL-M** 46.9 49.9 0.076 46.2 60.4 2.9*10^-11^

**LDL-S** 11.7 14.4 8.8*10^-5^ 11.4 18.8 1.3*10^-14^

**LDL-VS** 4.4 5.9 1.5*10^-6^ 4.3 7.1 1.1*10^-14^

**HDL-VL** 4.1 4.1 0.220 3.9 2.4 7.4*10^-11^

**HDL-L** 16.6 17.5 0.119 16.0 8.1 5.2*10^-13^

**HDL-M** 23.2 25.1 0.066 24.5 19.9 6.8*10^-13^

**HDL-S** 17.2 16.4 0.269 17.9 18.4 0.034

**HDL-VS** 5.0 5.5 5.1*10^-4^ 5.2 6.2 5.2*10^-10^

**VLDL-Size** 42.8 42.1 0.566 41.2 45.4 3.4*10^-11^

**LDL-Size** 26.22 26.15 0.136 26.24 26.10 9.8*10^-5^

**HDL-Size** 10.98 11.02 0.249 10.95 10.63 1.4*10^-13^

**ApoA1** 141.0 148.0 0.068 143.0 134.0 0.005

**ApoB** 70.5 82.0 0.005 66.5 98.6 7.0*10^-11^
